# Hyaluronic Acid Injections as Nonsurgical Alternative in case of Delayed Diagnosis of Malar Arch Fracture: Case Report and Literature Review

**DOI:** 10.1155/2019/1360741

**Published:** 2019-12-30

**Authors:** Raffaele Rauso, Giorgio Lo Giudice, Nicola Zerbinati, Gianpaolo Tartaro

**Affiliations:** ^1^Maxillo-Facial Surgery Department, University of Campania “Luigi Vanvitelli”, Naples, Italy; ^2^Maxillo-Facial Surgery, University of Naples “Federico II”, Italy; ^3^Dermatology Department, University of Insubria, Varese, Italy; ^4^Head of Maxillo-Facial Surgery Department, University of Campania “Luigi Vanvitelli”, Naples, Italy

## Abstract

**Background:**

In this article, we describe a nonsurgical approach based on hyaluronic acid filler injection to restore the facial features of a delayed diagnosis of malar fracture. We analyze the differences between surgical and nonsurgical solution: in case of early detection, the surgical approach is the gold standard of treatment. However, in cases of delayed facial fracture diagnosis without functional impairment, nonsurgical procedures could be considered an alternative tool as we show in the present case report.

**Aims:**

The aim of this study is to underline the importance of a complete aesthetic restoration in patients treated for noncosmetic purposes.

**Patients/Methods:**

We present a case of a 26-year-old male patient with a delayed diagnosis of malar fracture without functional impairment that was treated with hyaluronic acid (HA) filler injections.

**Results:**

The patient was followed up for 1 year showing stable results for the first 8 months; at the 12-month follow-up, a touch-up was suggested due to partial resorption of the filler.

**Conclusion:**

This is the first case describing a facial fracture treated with HA injections for only recontouring purposes. We assess that nonsurgical cosmetic procedures could be considered a “new” tool in the process of facial rehabilitation but only when functional problems are not associated with facial trauma.

## 1. Introduction

In the last 10 years, few papers have raised the attention to a most comprehensive aesthetic approach in facial restoration in case of congenital or acquired (trauma, tumor resection) anomalies [1–3]. WHO's definition of health, “a state of complete physical, mental and social well-being and not merely the absence of disease or infirmity,” clearly underlines how important is, especially for the facial area, a complete aesthetic restoration also in patients treated for noncosmetic purposes.

We present a case of delayed diagnosis of malar fracture without functional impairment treated with hyaluronic acid (HA) filler injections.

## 2. Case Report

**Figure 1:**
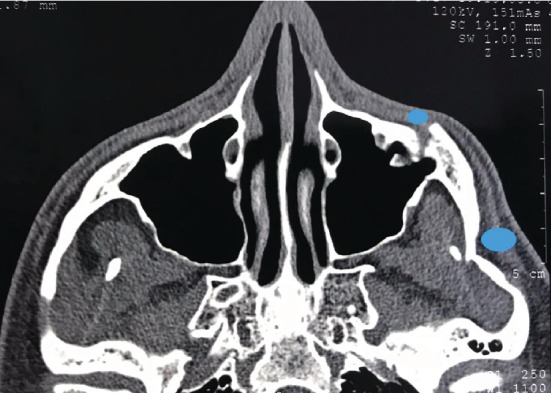
CT scan showing left displaced malar fracture. Blue dots point areas where HA was injected deep to the bone.

A 26-year-old male patient was referred to the Maxillo-Facial Unit at Campania's University Hospital 9 months after a facial trauma occurred during soccer match caused by collision with another player. After injury occurred, the patient experienced only swallowing without impairment in mouth opening and referred his symptom to a family doctor who suggested drug therapy with the aim to just reduce oedema. Only few weeks later, the patient noted facial asymmetry and a complete fracture of the zygomatic arch emerged after CT scan evaluation (Figure 1). Hence, the patient had several surgical counseling and he arrived at our hospital 9 months after trauma. We proceed immediately with a comprehensive clinical evaluation to exclude functional problems such as mouth opening reduction. A surgical approach and a nonsurgical approach were proposed to the patient: in the first case, a fracture of the poor consolidated malar bone, realignment and fixation with plate and wires of bone fragments was suggested; about the nonsurgical option proposed, it was based on the injections of HA filler to restore the proper projection of the traumatized side. Patient opted for nonsurgical options although it was clearly advised about the nonpermanent effect of the procedure.

**Figure 2:**
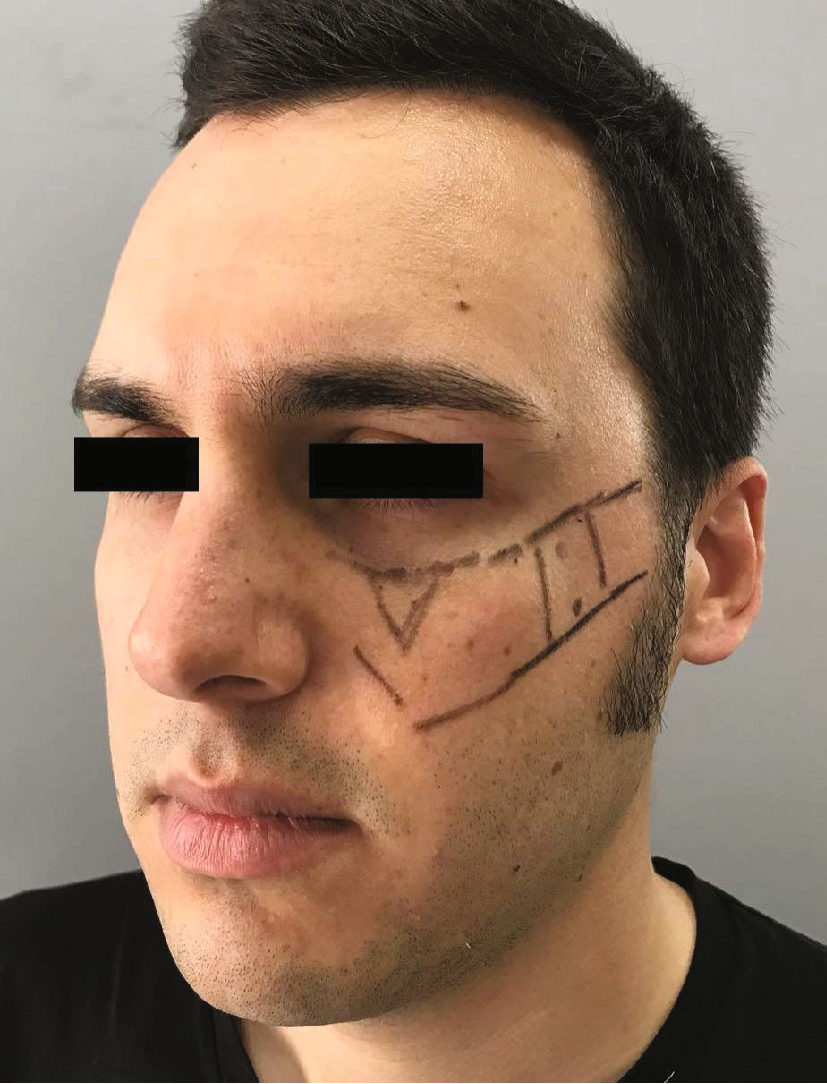
Preinjection marking.

**Figure 3:**
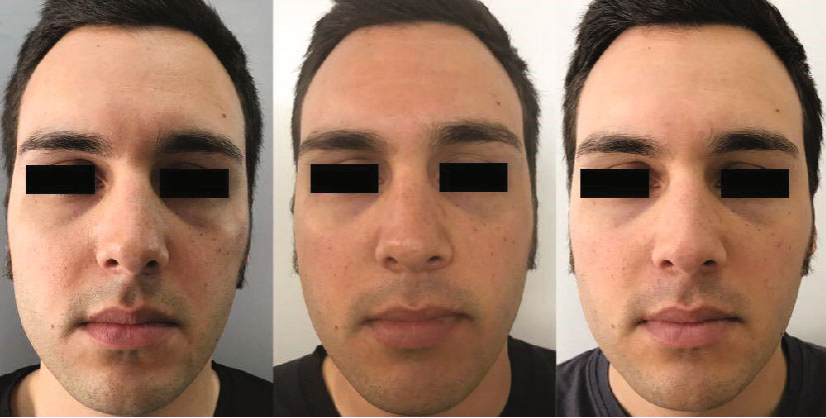
Preinjection and 2- and 5-month postinjection frontal view results.

**Figure 4:**
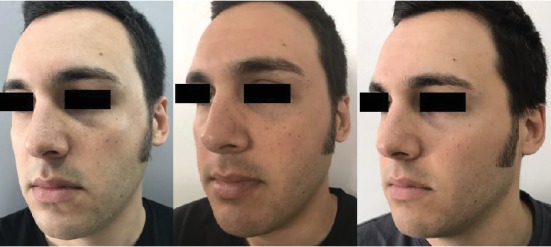
Preinjection and 2- and 5-month postinjection three-quarter left view results.

**Figure 5:**
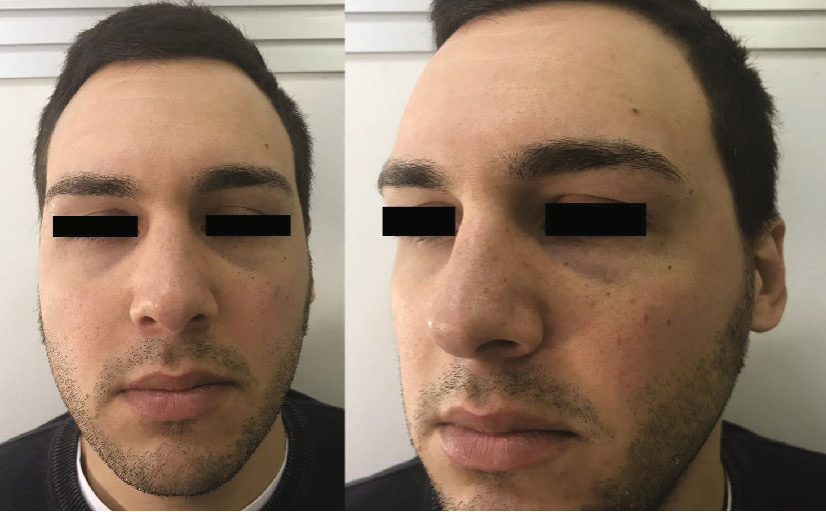
Frontal and three-quarter left view results at the 12-month follow-up.

After a careful evaluation of the CT scan, we decided to inject sovra-periosteally in the injured area: 1 mL of VYC-20L (Juvèderm Voluma with Lidocaine, Allergan plc, Dublin, Ireland) deep to the bone, 0.2 mL in the medial area, and 0.4 mL in two points in the lateral area (Figure 2); then, we used a cannula to restore the right malar projection injecting 1 mL of VYC-17L (Juvèderm Volift with Lidocaine, Allergan plc, Dublin, Ireland) in the subcutaneous layer. Patients did not experience ecchymosis nor oedema; no touch-up was required. The patient was followed up for 1 year showing stable results for the first 8 months (Figures 3 and 4); at the 12-month follow-up, a touch-up was suggested due to partial resorption of the filler (Figure 5).

## 3. Discussion

Aesthetic refinements with structural fat graft (SFG) following complex facial reconstructive surgery in case of posttraumatic facial soft tissues atrophy, tumor resection, congenital deformities, scleroderma, orbital and periorbital surgery, facial palsy, and burns have been largely described by Clauser et al. [3] with improvement in facial morphology, function, shape, and volume [3].

SFG is a surgical procedure standardized by Coleman and largely used for cosmetic and reconstructive purposes [4]. His primary indication was “to fill a gap,” although further studies have shown also a regenerative effect related to the preadipocytes present in the lipoaspirate [4]; also, nonsurgical cosmetic procedures, such as botulinum toxin type A and HA fillers, have been described to treat or improve symmetry in facial nerve injury [1].

In reviewing the existent literature, the following keywords were inserted on PubMed: filler AND facial trauma; filler AND facial fracture; non surgical treatment AND facial fracture; HA AND facial fracture; and hyaluronic acid AND facial fracture. The results are listed in Table 1. From the aforementioned PubMed search, all the articles were analyzed reading the abstract but no one spoke about the possibility to restore facial features with fillers after a facial fracture.

To the best of our knowledge, this is the first report of a facial fracture treated with a nonsurgical cosmetic procedure with the aim of malar contour symmetrization.

There are four main rheological parameters used to describe viscoelastic properties of a filler: G^∗^ (measures overall viscoelastic properties or “hardness”), G′ (measures elastic properties), G^″^ (measures viscous properties), and tan delta (measures the ratio between viscous and elastic properties).

G^∗^, the “complex modulus,” is the total energy needed to deform material using shear stress. This term is commonly referred as filler “hardness,” representing how difficult it is to alter the shape of an individual crosslinked unit of filler.

G′, the “storage/elastic modulus,” represents the energy fraction of G^∗^ stored by the gel during deformation and used to recover the original shape afterwards.

G^″^, the “loss/viscous modulus,” represents the energy fraction of G^∗^ lost on shear deformation through internal friction. G^″^ is not directly related to viscosity because HA filler is not purely viscous. Instead, this term reflects the inability of the gel to recover its shape completely after the shear stress is removed.

Tan delta refers to the elasticity of a material. Tan delta determines whether the material is mainly elastic (tan delta < 1), exhibiting a gel-like behavior (e.g., a block of gelatin), or whether it is mainly viscous (tan delta > 1), behaving more like a viscous liquid (e.g., honey).

In the present clinical case, we used two different types of filler: one with a higher G′ to restore the projection of the malaria area; the second one with a G′ lower than the first one but with a higher G^″^, so with an higher G^∗^, to recontour the subcutaneous malar layer.

The fillers used in the present study were Juvèderm Voluma with Lidocaine (VYC-20L; Allergan plc, Dublin, Ireland) and Juvèderm Volift with Lidocaine; both are hyaluronic acid–based fillers characterized by a mixture of low– and high–molecular weight HA chains (Vycross technology, Allergan) to improve moldability (ease of modelling/shaping), improve ease of flow during injection, reduce swelling of the gel within the tissue, improve distribution and integration within the tissue, and increase duration of effect [5]. All of these characteristics make VYC-20L and VYC-17L attractive candidates for our scope.

Although this is a nonpermanent solution for the patient, at the same time, a displaced malar fracture poorly consolidated after 9 months from the trauma requires a complex maxillofacial surgical procedure to restore midface eurhythmy with the necessity to perform new fractures to replace bone segments in the right position. Definitely in case of early detection and appropriate timing in malar fracture diagnosis, surgical procedure of bone replacement and fixation using plates and screws would have been the gold standard of treatment. It is well known that malar fracture needs to be recognized early after a trauma, because the oedema of the area can hide the fracture if it is not associated with functional limitation such as mouth opening reduction; however, a delayed surgical approach, in case of facial fracture, can be really challenging and, of course, can scare the patient who is just looking for aesthetic recontouring to face the malar depression secondary to the fracture.

HA facial fillers usually lasts about 6-8 months; however, in the present case, we noted a good long-lasting result; after 12 months from the injection, a slight reduction of malar arch projection was noted; however, the clinical situation was better compared to the preinjections. This long-lasting effect can be explained because deep HA injections, above the bones, were performed, and as showed by Mashiko et al., injecting HA deeply, there is an injury and persisting inflammatory changes around the injected HA particles that are expected to activate periosteal stem cells and contribute to the induction of tissue neogenesis, such as formation of capsule, fibrosis, and calcification/ossification during the HA absorption process [6]; this process could explain the long-lasting effect seen also in the present case, where the slight reduction of the projection of the area could be related to the resorption of the filler injected subcutaneously and not to the resorption of the one injected deep to the bone.

## 4. Conclusion

In the present case report, we describe a patient with tardive diagnosis of a poor consolidated broken fracture of the malar arch, without functional impairment, treated with HA injection to restore facial features. To the best of our knowledge, and after a PubMed search, this seems to be the first case describing a facial fracture treated with HA injections for only recontouring purposes. In our opinion, with the limitation of a case report, we assess that nonsurgical cosmetic procedures could be considered a “new” tool in the process of facial rehabilitation but only when functional problems are not associated with facial trauma and when the stigmata of the trauma have only a negative aesthetic impact.

## Figures and Tables

**Table 1:** The keywords searched on PubMed, through an abstract evaluation of the results, showed 0 match.

Keywords	No. of papers	Papers suitable for the research
filler AND facial trauma	135	0
filler AND facial fracture	3	0
non surgical treatment AND facial fracture	388	0
HA AND facial fracture	23	0
hyaluronic acid AND facial fracture	1	0
